# Measuring air layer volumes retained by submerged floating-ferns *Salvinia* and biomimetic superhydrophobic surfaces

**DOI:** 10.3762/bjnano.5.93

**Published:** 2014-06-10

**Authors:** Matthias J Mayser, Holger F Bohn, Meike Reker, Wilhelm Barthlott

**Affiliations:** 1 Microfluidics Lab, GRASP, University of Liege, Chemin des Chevreuils 1, 4000 Liege, Belgium; 2 Nees-Institute for Biodiversity of Plants, University Bonn, Venusbergweg 22, 53115 Bonn, Germany; 3 Plant Biomechanics Group Freiburg, University of Freiburg, Schänzlestrasse 1, 79104 Freiburg im Breisgau, Germany

**Keywords:** air layer, biomimetic, drag reduction, functional surfaces, plastron, Salvinia effect, volume measurement

## Abstract

Some plants and animals feature superhydrophobic surfaces capable of retaining a layer of air when submerged under water. Long-term air retaining surfaces (Salvinia-effect) are of high interest for biomimetic applications like drag reduction in ship coatings of up to 30%. Here we present a novel method for measuring air volumes and air loss under water. We recorded the buoyancy force of the air layer on leaf surfaces of four different *Salvinia* species and on one biomimetic surface using a highly sensitive custom made strain gauge force transducer setup. The volume of air held by a surface was quantified by comparing the buoyancy force of the specimen with and then without an air layer. Air volumes retained by the *Salvinia*-surfaces ranged between 0.15 and 1 L/m^2^ depending on differences in surface architecture. We verified the precision of the method by comparing the measured air volumes with theoretical volume calculations and could find a good agreement between both values. In this context we present techniques to calculate air volumes on surfaces with complex microstructures. The introduced method also allows to measure decrease or increase of air layers with high accuracy in real-time to understand dynamic processes.

## Introduction

Since the description of hierarchically structured, superhydrophobic, self-cleaning plant surfaces (Lotus-effect) [1–2] there has been an increasing interest in superhydrophobic surfaces [3–5]. Superhydrophobicity describes the extreme repellence of water by a surface. The level of water repellence is usually described by the contact angle which is the angle between the solid and the liquid at the three-phase contact line. Contact angles above 90° are considered hydrophobic while surfaces with contact angles above 150° are called superhydrophobic [6–9]. Smooth surfaces can reach a maximum contact angle of 120° [10]. Accordingly superhydrophobicity can only be achieved by a combination of a hydrophobic surface chemistry and surface structures on the micro and nano scale [11]. On these structured surfaces superhydrophobicity can occur either in the fully wetted state as described by Wenzel [12] or in the form of water sitting only on the tips of the surface structures (Cassie–Baxter wetting state) [13]. The contact angle of water droplets can be equally high in both wetting states [14–15]. However, in the Wenzel wetting state the water is in full contact with the surface and individual droplets adhere firmly [16]. In contrast to this in the Cassie wetting state the solid–water interface is strongly reduced while the majority of the interface is between water and air, thereby trapping an air layer between water and surface. As a result the adhesion of the water to the surface is minimised and individual droplets often roll off at very low tilting angles. However for true and persisting superhydrophobicity the Cassie wetting state has to be stable, i.e., no wetting transitions should occur [17–18]. One effective solution to prevent wetting transitions are surfaces with multiscale roughness [19–21]. Recently potential applications for these trapped air layers in the Cassie wetting regime have been proposed which include drag reducing ship coatings or fluid channels [22–26] with the capability of 30% drag reduction [27] and could provide high economic and ecologic value [28–29].

**Figure 1:**
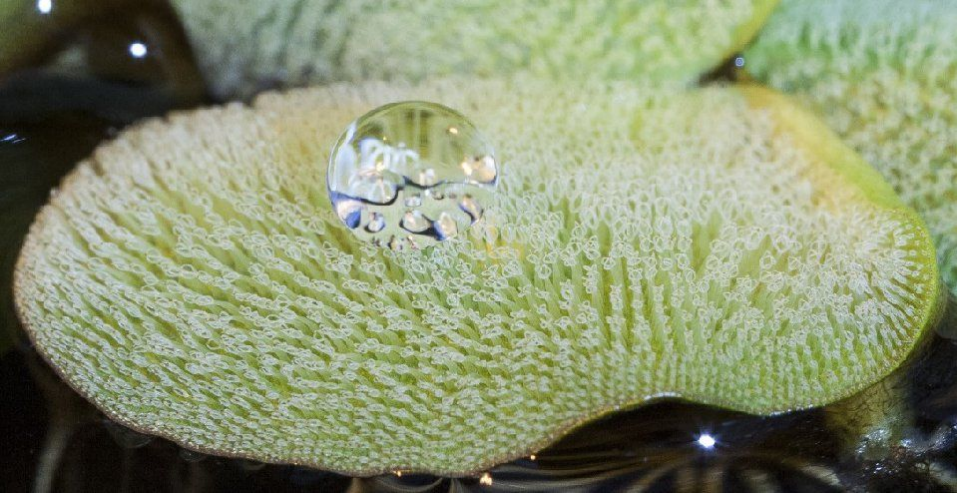
Leaf of *Salvinia molesta* floating on water. The leaf surface is densely covered with complex superhydrophobic “egg-beater” hairs. The height of the hairs decreases towards the margin of the leaf. An applied water droplet resides on the tips of the hairs without sinking between the hairs.

While superhydrophobic plant surfaces, e.g., the leaves of Lotus (*Nelumbo nucifera*) provide very high contact angles and low hysteresis [1], the air layers that are held between the surface structures persist only for short periods of time [22]. However, for some biological surfaces like the elytra of the back swimmer *Notonecta* or the upper leaf side of floating ferns of the genus *Salvinia* air layers are reported to persist from several days up to months [30–32]. Responsible for the long-term air retention in these organisms is a dense cover of elaborate, hydrophobic hairs on their surfaces (Figure 1). In *Salvinia* these hairs possess a multiscale roughness on several hierarchical levels resulting in a stable Cassie wetting state [17].

In the floating fern genus *Salvinia* four different shapes of such hairs (trichomes) have been described [33]. In the simplest case these are individual multicellular, uniseriate trichomes. In the most complex case four multicellular, uniseriate trichomes are grouped on top of an emergence and join at their tips forming a so called “egg beater” hair. These hairs with heights between approximately 300 and 2200 µm cover the major part of the upper leaf side (Figure 1). Towards the margin of the leaf the height of the hairs decreases gradually in a smooth transition (Figure 1) [33]. This 'edge effect' is probably responsible to maintain an entire air layer over the leaf. Both the hairs and the remaining cell surface are hydrophobically covered with waxes in the shape of thin rodlets perpendicular to the surface [33–35]. As special characteristic of *S. molesta* the topmost cells of its hairs lack the wax cover and are thereby hydrophilic while the remaining part is hydrophobic. The resulting pinning of the water to these hydrophilic tips 'Salvinia-effect' has been proven to increase the stability of the air layer under low pressure conditions (e.g., turbulences) and prevent the extraction of air large bubbles [35–36].

Though the air layers held by *Salvinia* leaves have previously been studied qualitatively, the amount of air, held in these layers, has not been quantified. In this study we present a new method to measure the volume of air retained by biological and artificial superhydrophobic surfaces quantitatively. Air layers of four *Salvinia* species and of well defined replicas were analysed.

## Results and Discussion

### Air volume on wafer replica

For the validation of the method we use the microstructured wafer replicas as a defined control surface and compare the measured values with the calculated theoretical air volumes.

The theoretical air volume held by the wafer is determined by the dimension of the microstructured area subtracted by the volume occupied by the pillars. Thus the proportion of air per total surface area (*i*_A_) is

[1]
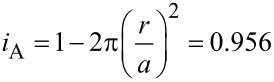


with *r* being the pillar radius and *a* the diagonal pitch of the pillars.

The structured area has a length *l* of 18.8 mm, a width *w* of 14 mm and 60 µm pillar height *h*. Assuming a smooth air–water interface the theoretical air volume *V*_A_ can be calculated to

[2]
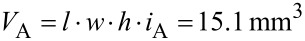


The air volume held by the microstructured wafer replicas was measured to a mean value of 14.8 ± 0.3 µL (*n* = 10). This is approximately 2% below the theoretical air volume, which also lies within the standard deviation of the measured data. The slightly lower values might be explained by the shape of the air water interface, which is not smooth but sagging in between the pillars, so that the real air volume should be slightly smaller than the theoretical value [37]. Another reason could be defects in the hydrophobic coating, leading to small areas, where there might have happened a transition to the Wenzel wetting state thereby reducing the air volume of the sample. In two cases the measured values were higher than the calculated one. These higher air volumes were probably caused by small air pockets on top of the structured surface or bubbles below the sample, which could appear if the replica was submerged to fast.

### Air volumes on *Salvinia* leaves

**Figure 2:**
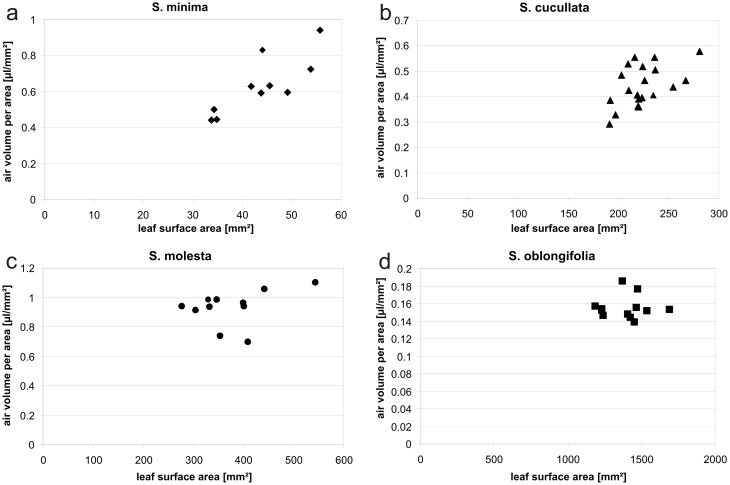
Air volume per surface area measured on four different *Salvinia* species. a) *S. minima* (*n* = 10), b) *S. cucullata* (*n* = 19), c) *S. molesta* (*n* = 11), d) *S. oblongifolia* (*n* = 12).

The air volumes held by four different *Salvinia* species were more variable due to the more complex surface microstructure (see Figure 4) and great differences in leaf surface area which ranged from 44 ± 8 mm^2^ for *S. minima* up to 1388 ± 149 mm^2^ for *S. oblongifolia* (*S. cucullata*: 224 ± 23 mm^2^, *S. molesta* 359 ± 52 mm^2^). For better comparability of the different species we normalised the measured air volumes with the leaf surface area (Figure 2). In order to test whether the leaf surface area has an influence on the air volume per surface area we performed a linear correlation analysis. For the species with small leaves, *S. minima* (*r*^2^ = 0.665, *p* ≤ 0.05) and *S. cucullata* (*r*^2^ = 0.273, *p* ≤ 0.05) a significant correlation was found. In the species with larger leaves, *S. molesta* (*r*^2^ = 0, *p* = 0.992) and *S. oblongifolia* (*r*^2^ = 0, *p* = 0.971) an effect of the overall leaf surface area on the air volume per surface area could not be found. As the trichomes decrease in height towards the leaf edges, the air volume per leaf area also decreases towards the leaf edges. Idealising a leaf as a circle, the edge length (circumference) rises linearly with radius (*C* = 2π*r*) while there is a quadratic increase for the leaf surface area (*A* = π*r*^2^). Accordingly the decreasing air volumes towards the edges of the leaves have a larger influence on the overall air volume on small leaves of *S. minima* and *S. cucullata* (Figure 2a,b), than they have in species with larger leaves like *S. molesta* and *S. oblongifolia* (Figure 2d).

For the theoretical volume calculation we assume a circular leaf (radius *R*) with a linear decline of the trichome height *H* towards the edge for a width *W* specified for each species (see Table 1). This results in an air layer in the shape of a truncated cone. The volume of this truncated cone represents the maximum theoretical volume *V*_max_ of an air layer on a leaf with a given surface area and can be calculated to

[3]
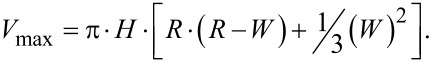


Divided by the overall surface area, this results in the mean air height. For an infinitely large leaf, where the decrease of trichome height at the edge is negligible, this should almost equal the trichome height on the plants.

**Table 1:** Dimensions of characteristic trichome-parameters of four different *Salvinia* species (mean ± s.d., *n* = 10). For details see Figure 4.

			*S. oblongifolia*	*S. cucullata*	*S. minima*	*S. molesta*

Trichome density	ρ	1/mm²	25.8 ± 3.3	13.0 ± 2.6	2.25 ± 0.23	1.64 ± 0.24
Trichome height	*H*	µm	310 ± 41	558 ± 143	919 ± 107	2.629 ± 285
Trichome length	*L*	µm	370 ± 46	609 ± 152	995 ± 108	2.922 ± 264
Emergence length	*l*_E_	µm	177 ± 30	n.a.	443 ± 93	1.955 ± 302
Egg beater shape length	*l*_C_	µm	n.a.	n.a.	n.a.	967 ± 51
Hair length	*l*_H_	µm	222 ± 28	609 ± 152	562 ± 46	1160 ± 88
Emergence diameter (base)	*d*_Eb_	µm	269.0 ± 8.6	n.a.	287 ± 18	590 ± 45
Emergence diameter (tip)	*d*_Et_	µm	168 ± 13	n.a.	139 ± 11	177 ± 13
Egg beater hair diameter	*d*_C_	µm	n.a.	n.a.	n.a.	613 ± 73
Hair diameter (base)	*d*_Hb_	µm	68.5 ± 6.8	40.5 ± 6.2	71.2 ± 6.9	70.9 ± 6.7
Hair diameter (tip)	*d*_Ht_	µm	26.5 ± 4.8	9.6 ± 1.3	20.9 ± 3.0	n.a.
Width of leaf edge	*W*	µm	428 ± 80	1639 ± 205	966 ± 143	3292 ± 321

**Figure 3:**
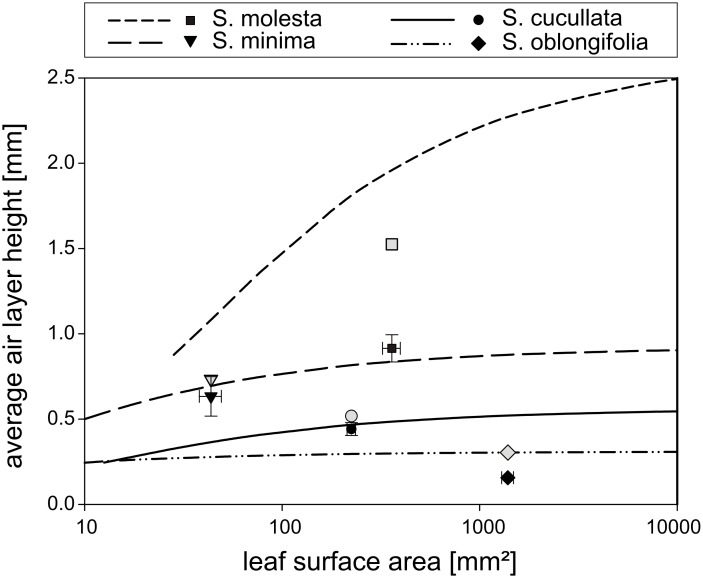
Air volume per surface area measured on four different *Salvinia* species compared to the calculated theoretically maximum values. (symbols: median values, whiskers: 95% confidence interval, grey symbols: measured values with calculated values for hair volume and air–water interface sagging added, lines: theoretically maximum values).

Comparing the theoretical values with the values measured on *Salvinia* leaves shows different results for the different species (Figure 3). All measured values are below the calculated theoretical ones. Most of these differences can be explained by the volume occupied by the trichomes themselves and by the sagging of the air water interface between the hairs. Since the surface microstructure of the *Salvinia* leaves is less well defined than in the wafer replicas, these volumes are difficult to measure but can be approximated. Table 1 displays the measured surface structure dimensions and parameters for the four investigated *Salvinia* species needed for this calculation. To estimate the volume occupied by the trichomes, we idealized them as assemblies of truncated cones for the emergence and the hairs. The volumes of these truncated cones were calculated with the measured mean dimensions (see Table 1), summed up to represent an individual trichome and multiplied by the trichome density ρ to get a volume per surface area (Table 2).

**Table 2:** Calculated trichome volumes.

		*S. oblongifolia*	*S. cucullata*	*S. minima*	*S. molesta*

Volume (hairs)	nL	2 × 0.42	0.34	4 × 1.03	4 × 2.08
Volume (emergence)	nL	4.80	n.a.	16.40	241.23
Volume (trichome)	nL	5.64	0.34	20.52	249.57
Trichome volume per surface area	nL/mm^2^	145	4	46	409

Additionally, to estimate the volume of the water sagging in between the hairs the shape of the air–water interface is transferred to a solid material by a replication technique (see 'Characterisation of the air–water interface'). On these replicas the depth and width of the sagging was measured (Table 2). The indentions of the replicated air–water interfaces were approximated as ball scrapers with the measured width *d* and height *h*. Their volume *V*_BS_ was calculated as

[4]
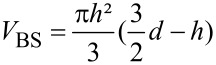


and then again multiplied with the trichome density ρ to get a volume per surface area (Table 3).

**Table 3:** Measurement values and volume calculations of the water sagging in between the hairs (mean ± s.d., *n* = 10).

			*S. oblongifolia*	*S. cucullata*	*S. minima*	*S. molesta*

Width of sagging	*d*	µm	193 ± 36	511 ± 159	727 ± 245	1224 ± 148
Height of sagging	*h*	µm	20.7 ± 9.2	75.0 ± 18.4	72.5 ± 17.3	187.0 ± 60.8
Volume of ball scraper	*V*_BS_	nL	0.3	7.0	14.4	99.7
Volume of sagging per surface area		nL/mm^2^	7.5	91.0	32.4	163.5

When both values are added to the measured air volumes per surface area the results coincide well with the theoretical volumes except for *S. molesta* (grey symbols in Figure 3). For the other three species the new values are all slightly above the measured air volumes (Table 4). This is due to the hairs bending towards the leaf surface when submerged because of the static water pressure thereby decreasing the volume of the trapped air. During the size measurements the hairs were fully erect, which results in a higher air volume in the calculations. However, for the low submerging depth used in the experiments these differences are very small (Table 4).

**Table 4:** Difference between the measured air volumes and the calculated theoretically maximum values in percent with and without the hair volumes and the shape of the air–water interface taken into account.

		*S. oblongifolia*	*S. cucullata*	*S. minima*	*S. molesta*

Difference without hair volume and air–water interface influence	%	−48.7	−5.4	−10.5	−53.3
Difference with hair volume and air–water interface influence	%	+4.3	+7.1	+3.8	−25.6

*S. molesta* leaves feature a different leaf form, which consist of two halves joined only in a small portion in the middle and having a rather pointed angle between them. Though the experiments were performed with care to prise the two halves apart, in a portion of the leaf surface near the joint the hair tips would interlink between each other and form an air pocket, which is considerably smaller than an air film with full height on those surfaces. Still the correction of the measured volumes with the hair volumes and air–water interface shape influence reduced the difference between this value and the theoretically calculated one by more than the factor of two (Table 4).

## Conclusion

We have presented a reliable method for precisely measuring volumes of air layers on submerged superhydrophobic surfaces. With the current set-up air volumes up to 900 µL can be measured with a precision of 0.1 µL. By adjusting the lever length of the needle the measurement range and resolution can be tuned appropriate to the sample and air layer volume. Apart from technical surfaces the method has been successfully applied to measure air volumes held by living biological samples. It was shown that the investigated *Salvinia* species with their highly complex hierarchically structured surfaces hold up to 1 L/m^2^ air under water.

Furthermore we demonstrated a method for taking measures of the air–water interface by replicating its shape into a solid material. This in combination with the calculation of the volume of the surface structures allowed us to correlate the measured air volumes with theoretically estimations. The combination of these two techniques allows the precise measurement of air layer heights even if the dimensions of the surface structures are not predefined like on artificial surfaces.

We recently used the method to analyse the long term behaviour of air layers, as it is capable of measuring changes in buoyancy in real-time over extended periods, thereby giving an insight in the dynamic persistence of air layers and the progresses of their decrease (publication in preparation). This knowledge about the dynamic behaviour of air layers can be fundamental for the creation of artificial surfaces with long-term air retention. Recent measurements by our project partners have revealed a drag reduction of 30% by such air retaining surfaces [27]. Applied on ships this would have a large impact on fuel consumption.

## Experimental

### Materials

**Figure 4:**
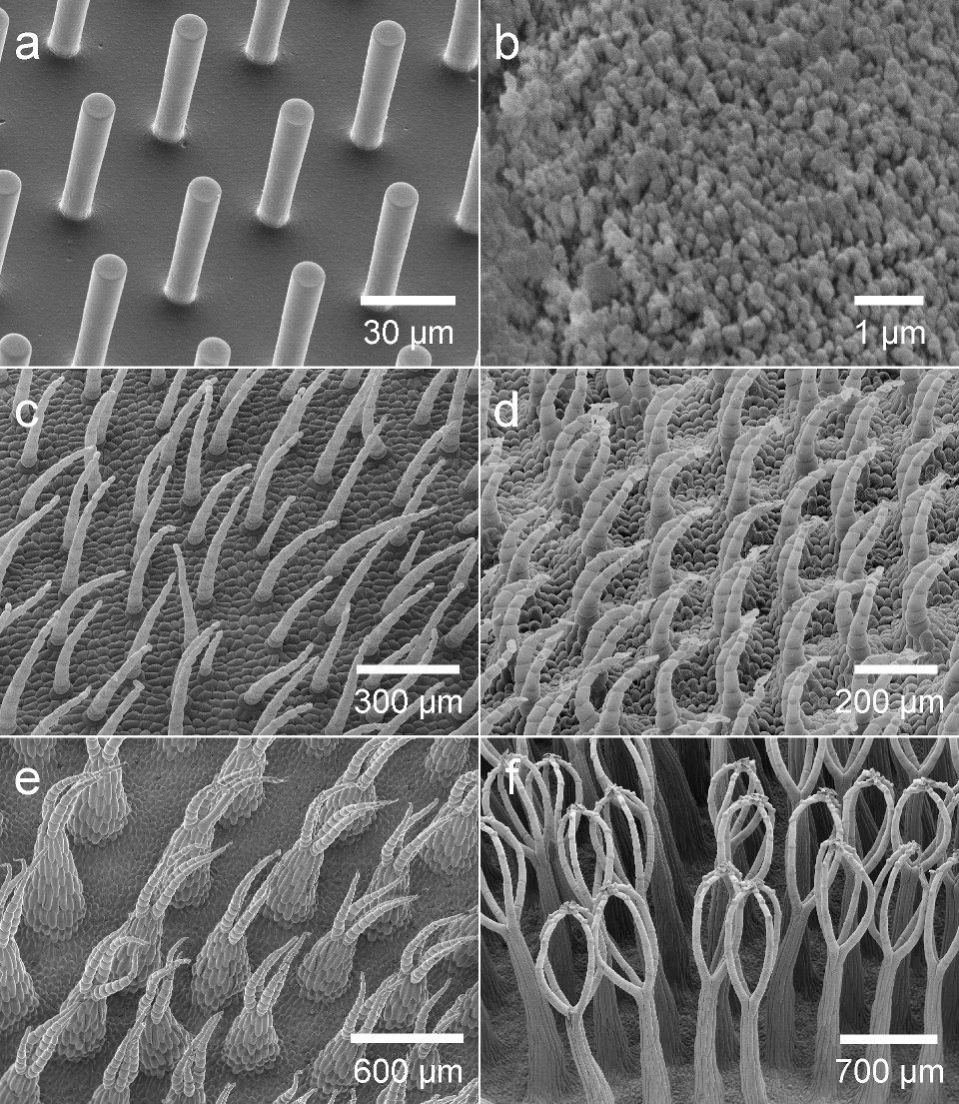
SEM images of technical and biological microstructured surfaces used for air volume measurements. a) uncoated epoxy-resin replica of a silicon wafer with pillars of 10 µm diameter and 60 diagonal pitch, b) hydrophobic coating of the replicas with Tegotop 210 in high magnification, c–f) upper leaf surface of four *Salvinia* species c) *Salvinia cucullata*, d) *S. oblongifolia*, e) *S. minima*, f) *S. molesta*.

In order to test the method with a well-defined, structured surface, a silicon waver (18.8 × 14 mm) with pillars of 60 µm height, 10 µm diameter and a rectangular pattern with 60 µm diagonal pitch (Figure 4a) were replicated with epoxy resin (Injection Resin EP, Reckli GmbH, Germany) as described by Koch et al. [38]. Replicas were hydrophobically coated with 25 µL of diluted (1:10) Tegotop 210 (Evonik Degussa GmbH, Germany) (Figure 4b).

Fresh leaves of four different *Salvinia* species representing all described trichome types [33] were measured. *S. cucullata* (BG BONN 18268) displays simple, slightly curved hairs, consisting of 6 to 8 cells in a row (Figure 4c). *S. oblongifolia* (BG BONN 14457) incorporates trichomes which consist of two hairs, which grow out of a slight emergence and are joined at their topmost cells (Figure 4d). The Natans-type represented by *S. minima* (BG BONN 8595) shows four hairs growing on top of a large emergence which are not joined at their tips (Figure 4e). The so called “egg beater" hairs on *S. molesta* (BG BONN 14459) consist of a large emergence topped with four hairs which are joined at their tips (Figure 4f). Plants were cultivated in the Botanical Gardens of the University of Bonn.

### Buoyancy measurement

**Figure 5:**
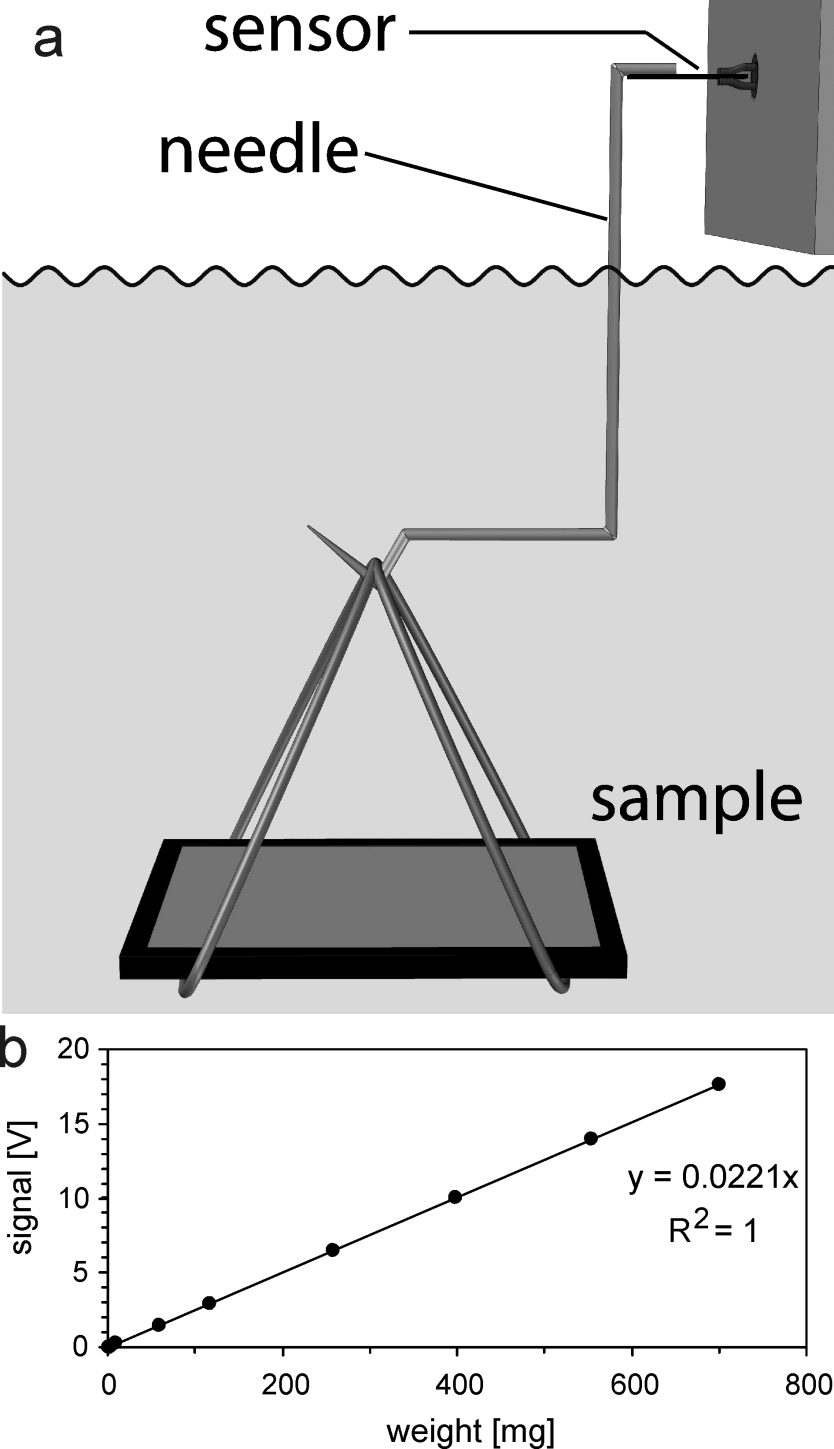
Buoyancy measurement setup. a) Schematic drawing of the measurement setup. A bent metal needle is glued to a silicon strain gauge sensor and submerged under water. Specimens with air layer were hung onto the needle by nylon strings. b) Calibration curve of the force transducer using nine weights in the range between 1 and 700 mg.

The volume of air retained by a surface was determined by measuring the buoyancy force the air generates. This was accomplished by installing a force transducer consisting of a silicon bending beam with integrated strain gauge (Sensor Element AE801, HJK, Germany) right above the water level in an aquarium filled with water. A metal needle (40 mm length, 0.4 mm diameter, V2A steel) was bent into an L-shape and glued to the end of the beam with resin glue resulting in the tip of the needle being submerged 20 mm below the water surface. All specimens were mounted to the force sensor by hanging them into a bend close to the tip of the needle with nylon strings, thereby ensuring a fixed lever arm between the applied weight and the strain gauge (Figure 5a). The change in resistance generated through the bending of the beam by buoyancy forces of the attached specimen was measured by means of a Wheatstone bridge, an amplifier (G1T8, ME Meßsysteme GmbH, Germany) and a A/D converter (NI-USB6009, National Instruments, USA). The recorded voltages were converted into forces through a calibration coefficient acquired with a set of nine weights in the range from 1 to 700 mg. The calibration measurements were performed in air prior to the experiment. The calculated coefficient was 45.25 mg/V with a highly linear behaviour across the whole measurement range (Figure 5b). The buoyancy force of air under water equals the weight difference to the displaced water; accordingly 45.25 mg/V equals 45.25 µL of submerged air per Volt assuming a water density of 1 mg/µL. The maximum sensitivity of the measurement was 0.09 µL.

For proper mounting to the force sensor specimens had to possess negative buoyancy when submerged under water. Thus, *Salvinia* leaves along with additional weights were glued onto a rectangular plastic straps using a polyvinylsiloxane (President Light Body, ISO 4823, Coltene Whaledent, Hamburg, Germany). Wafer replicas showed negative buoyancy without further modification. Wafers and mounted leaves were attached to two slings of nylon string (0.1 mm diameter), with which they were then hung into the bend of the needle. The samples were thereby held at approximately 80 mm below the water surface during the experiment. For the measurement a sample with an air layer was attached to the needle and given 10 seconds to stabilise before the buoyancy was recorded (mean value over 10 seconds after stabilising time). Then the sample was detached from the needle, taken out of the water and covered with 70% ethanol for wetting the surface. Afterwards it was washed for 20 seconds with water and then reattached to the needle for renewed buoyancy measurement. From the difference in buoyancy before and after removal of the air layer its volume was calculated.

### Surface characteristics of *Salvinia* and replicas

The surface microstructures of both technical and biological surfaces were visualised by scanning electron microscopy (SEM). The wafer replicas were coated with gold by a sputter coater (SCD-040, Balzers Union, Liechtenstein) at 60 mA for 30 seconds before SEM investigation. The plant leaves were prepared for SEM examination by dehydration in alcohol and ‘critical point drying’ (CPD 020, Balzers Union, Liechtenstein) and also gold coated at 15 mA for 120 seconds. Electron microscopy was performed on a Cambridge Stereoscan 200 SEM (Zeiss GmbH, Oberkochen, Germany) with digital image processing unit (DISS 5, Point electronic GmbH, Halle, Germany) at acceleration voltages of 15 kV for the replicas and 10 kV for the plant material.

The leaf surface area was acquired after the volume measurement by scanning the flattened leaf with a flat bed scanner (Canon 4200F, Canon Inc., Japan) and measuring the leaf area on the image according to a calibration grid.

**Figure 6:**
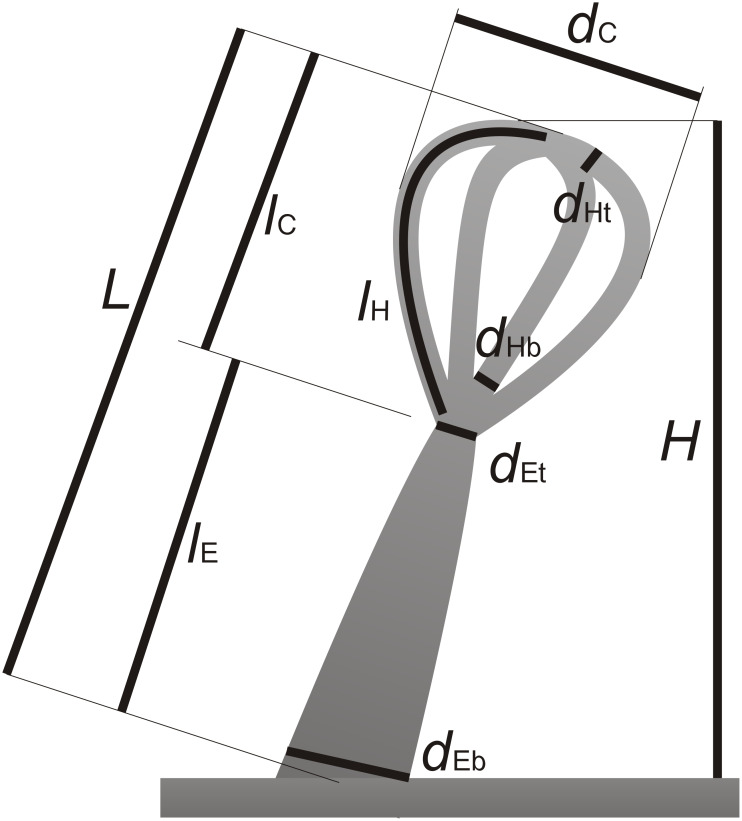
Schematic drawing showing the structural parameters acquired from microscopic images of *Salvinia* leaf surfaces: trichome height *H*, trichome length *L*, emergence length *l*_E_, egg beater shape length *l*_C_, hair length *l*_H_, emergence base diameter *d*_Eb_, emergence tip diameter *d*_Et_, egg beater shape diameter *d*_C_, hair base diameter *d*_Hb_, hair tip diameter *d*_Ht_.

A digital light microscope (VHX-1000, Keyence, Japan) was used to measure the trichome dimensions of the examined *Salvinia* species. Images of fresh leaves were taken from straight above and sideward in order to measure width and diameter of the trichomes as well as the trichome density (Figure 6). All parameters were taken at the centre of leaf halves (where the hairs are highest) and are given as mean values with standard deviations of *n* = 10.

### Characterisation of the air–water interface

**Figure 7:**
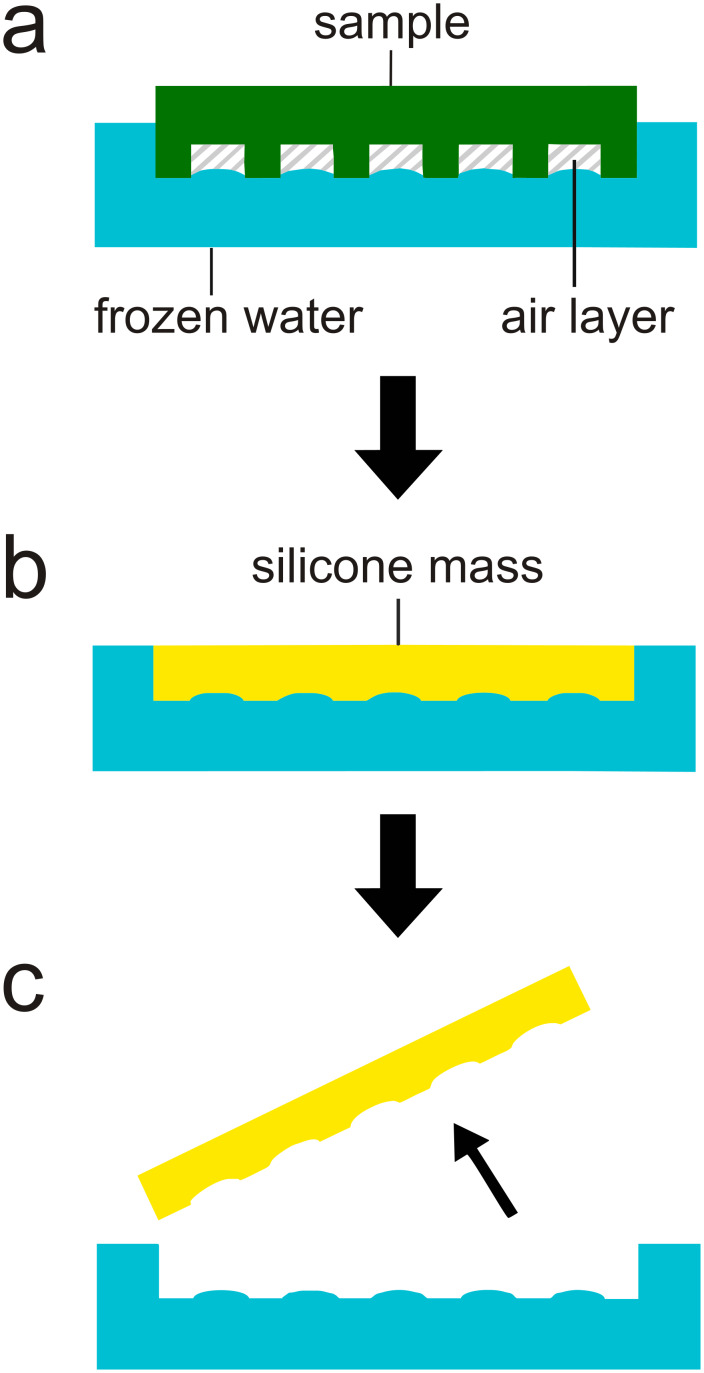
Scheme of the replication of the air–water interface. a) A *Salvinia* leaf is placed upside down on water and the assembly is cooled to −32 °C afterwards. b) After removing the leaf specimen the imprint in the frozen water was filled with cooled silicone. c) After two days the polymerized silicone was removed.

The air–water interface on the *Salvinia* leaves was replicated into a solid material to be able to measure it and estimate the volume of air displaced by the water sagging in between the hairs. For this purpose individual leaves were attached to the inside of the lid of a standard Petri dish using super glue. The bottom part of the Petri dish was filled with water and afterwards the lid with the attached leaf was placed on top, resulting in the leaf surface just penetrating the water surface inside the Petri dish. The leaf edge was kept out of the water to establish a connection between the air layer and the surrounding air. This prevented the air layer to reduce in volume during cooling, which would have changed the shape of the air–water interface. The assembly was then cooled to −32 °C. The Petri dish with the frozen water was placed in a box with solid CO_2_ and the leaf was pulled out of the ice by lifting the lid of the Petri dish. The solid CO_2_ would serve a dual purpose of maintaining cold temperatures and providing a CO_2_ atmosphere to prevent humidity from condensing on the frozen air–water interface. The indention in the ice was filled with silicone (Honigum®, DMG Chemisch-Pharmazeutische Fabrik GmbH, Hamburg, Deutschland, ISO 4823:2000, Type 3: Light-bodied consistency) which was cooled below 0 °C previously. The CO_2_ prevented crystallisation of humidity on the cool surface. After hardening at −32 °C for 2 days the silicone was removed from the ice and represented the shape of the air–water interface of the air layer under water (Figure 7). This silicone replica was examined by an optical 3D microscope (Modell VHX-1000, Keyence Corporation, Osaka, Japan) and the depth and width of the sagging was measured.
